# Evasion of Intracellular DNA Sensing by Human Herpesviruses

**DOI:** 10.3389/fcimb.2021.647992

**Published:** 2021-03-15

**Authors:** Debipreeta Bhowmik, Fanxiu Zhu

**Affiliations:** Department of Biological Science, Florida State University, Tallahassee, FL, United States

**Keywords:** cGAS, DNA sensing, innate immune response, herpesvirus, viral evasion

## Abstract

Sensing of viral constituents is the first and critical step in the host innate immune defense against viruses. In mammalian cells, there are a variety of pathogen recognition receptors (PRRs) that detect diverse pathogen-associated molecular patterns (PAMPs) including viral RNA and DNA. In the past decade, a number of host DNA sensors have been discovered and the underlying sensing mechanisms have been elucidated. Herpesviruses belong to a large family of enveloped DNA viruses. They are successful pathogens whose elaborate immune evasion mechanisms contribute to high prevalence of infection among their hosts. The three subfamilies of herpesviruses have all been found to employ diverse and overlapping strategies to interfere with host DNA sensing. These strategies include masking viral DNA or the DNA sensor, degradation of the DNA sensor, and post-transcriptional modification of the DNA sensor or its adaptor protein. In this review, we will discuss the current state of our knowledge on how human herpesviruses use these strategies to evade DNA-induced immune responses. Comprehensive understanding of herpesvirus immune-evasion mechanisms will aid in the development of vaccines and antivirals for herpesvirus-associated diseases.

## Introduction

Herpesviruses belong to a family of large DNA viruses that characteristically cause both latent and lytic infections in a wide range of animals and humans. There are eight types of herpesviruses currently known to infect humans, causing various diseases (Knipe and Howley, 2013). Herpesviruses are subdivided into the α-, β-, and γ-subfamilies, based on biologic properties including cell tropism, genome organization, and sequence homologies of the conserved open-reading frames (ORFs) (Davison et al., 2009) (**Table 1**).

**Table 1:** Classification of human herpesviruses.

Subfamily	Type	Synonym	Cytopathology	Primary target cells	Site of latency	Pathophysiology
α-herpesvirus	HHV-1	Herpes simplex virus-1 (HSV-1)	Cytolytic	Mucoepithelial	Neurons	Orofacial infections, Encephalitis
	HHV-2	Herpes simplex virus-2 (HSV-2)				Genital and neonatal infections
	HHV-3	Varicella zoster virus (VZV)				Chickenpox, Shingles
β-herpesvirus	HHV-5	Cytomegalovirus (CMV)	Cytomegalic	Monocytes, lymphocytes, epithelial cells	Macrophages, lymphocytes, epithelial cells	Congenital infection, Sensorineural hearing loss in children, Retinitis, Hepatitis
	HHV-6	Roseolovirus	Lymphotropic	T cells	Monocytes/macrophages	Exanthem subitum
	HHV-7	Roseolovirus		T cells	T cells	Roseola infantum
γ-herpesvirus	HHV-4	Epstein–Barr virus (EBV)	Lymphoproliferative	B cells and epithelial cells	B cells	Infectious mononucleosis, lymphoma, carcinoma
	HHV-8	Kaposi’s sarcoma-associated herpesvirus (KSHV)		Lymphocytes and endothelial cells	Lymphocytes	Kaposi’s sarcoma, Primary effusion lymphoma, Multicentric Castleman disease

The host innate immune response is the first line of defense against viral infection. It is activated by the host pattern recognition receptors (PRRs) upon detecting conserved molecular moieties from the pathogens, known as Pathogen Associated Molecular Pattern (PAMPs) (Mogensen, 2009). Typical PAMPs include lipopolysaccharides of bacteria that can be recognized by Toll-Like Receptor (TLR)4, and dsRNA of viruses that can be detected by TLR3 in endosome, retinoic acid-inducible gene I (RIG-I), and melanoma differentiation-associated gene 5 (MDA5) in the cytosol. Despite being the genetic blueprint of all life, DNA has long been known to be immune stimulatory (Isaacs et al., 1963). As a potent PAMP, DNA can be recognized by several PRRs (**Table 2**) (Paludan and Bowie, 2013; Unterholzner, 2013; Dempsey and Bowie, 2015; Ma et al., 2018). The first described DNA sensor that detects endosomal CpG-rich DNA to initiate type I interferon (IFN) response was TLR9 (Hemmi et al., 2000). Soon after, Absent In Melanoma 2 (AIM2) was found to sense cytosolic DNA and trigger inflammasome response (Fernandes-Alnemri et al., 2009; Hornung et al., 2009). Several other DNA sensors like IFNγ-Inducible protein 16 (IFI16), RNA polymerase III (Pol III), and the Mre11-Rad50-Nbs1 (MRN) complex, have also been reported (Goubau et al., 2013; Paludan, 2015; Volkman et al., 2019). The most recently reported DNA sensor is cyclic GMP–AMP Synthase (cGAS) which was discovered by Dr. Zhijian “James” Chen’s group through elegant biochemical approaches (Sun et al., 2013; Wu et al., 2013). Binding to dsDNA activates cGAS, leading to generation of unique second messenger cGAMP that binds to stimulator of interferon genes (STING) and activates downstream signaling (Ablasser et al., 2013; Gao et al., 2013; Sun et al., 2013). Numerous studies have shown cGAS to be the non-redundant principal cytosolic DNA sensor in most cells. All PRRs share similar modes of action: after the recognition of PAMPs, PRRs induce intracellular signaling pathways through the hierarchical activation of a PRR family-specific adaptor protein. This leads to the expression of genes with pro-inflammatory and microbicidal activities, including cytokines and type I IFNs. Secreted cytokines and chemokines are also critical for shaping effective adaptive immune responses (Akira et al., 2006; Brubaker et al., 2015).

**Table 2:** Characterization of the DNA sensing pathways.

Proposed DNA sensor	Ligand	Cite of DNA sensing	Mechanism	Biological Response	References
**Toll-like receptor**					
TLR9	CpG DNA	Endosomes	Recognizes unmethylated CpG DNA and recruits the adaptor protein MyD88 to induce activation of NF-κB and IRF7	Type I IFN	Kawai and Akira, 2011
**PYHIN Family**					
AIM2	Cytosolic dsDNA	Cytoplasm	Binds to DNA *via* AIM2 HIN200 domain and recruits ASC *via* a pyrin:pyrin homotypic interaction followed by subsequent caspase activation	IL-1β and IL-18	Hornung et al., 2009
IFI16	Cytosolic dsDNA Nuclear dsDNA	Cytoplasm, nucleus	1. Interacts with STING, and subsequently induces IRF-3 phosphorylation. 2. Activates inflammasome responses through ASC and caspase-1	IFN-β, IL-6, IL-1β	Kerur et al., 2011; Ansari et al., 2013; Johnson et al., 2013 Ansari et al., 2015
**Nucleotidyl Transferase Family**					
cGAS	Cytosolic dsDNA Y-form DNA DNA-RNA hybrid	Cytoplasm, nucleus	Binds to DNA and catalyzes the synthesis of cGAMP that activate STING followed by subsequent activation of IRF3	IFN-β	Ablasser et al., 2013; Gao et al., 2013; Sun et al., 2013
**Protein Kinase (PK) Family**					
DNA-PK	Cytosolic dsDNA	Cytoplasm	1. Senses DNA and activates the STING-TBK1-IRF3 axis 2. Induces STING independent DNA sensing pathway by triggering HSPA8 and IRF3 phosphorylation	IFN-λl, IFN-β, IL-6	Burleigh et al., 2020
Ku70	Cytosolic dsDNA	Cytoplasm	Senses DNA and induces the production of type III IFN	Type III IFN	Zhang et al., 2011
**DExD/H-Box Helicase Family**					
DHX9** **and DHX36	CpG DNA	Cytoplasm	Detect CpG DNA and activate an MyD88-dependent pathway	TNF-α, IFN-α	Kim et al., 2010
**Other DNA sensors**					
DAI	Cytosolic dsDNA	Cytoplasm	Senses DNA and activates the STING-TBK1-IRF3 axis	IFN-β	Takaoka et al., 2007
Mre11	Cytosolic dsDNA	Cytoplasm	Senses DNA and activates the STING-TBK1-IRF3 axis	IFN-β	Kondo et al., 2013

To overcome host defenses, viruses, including herpesviruses, have evolved multiple strategies to evade immune recognition by PRRs (Bowie and Unterholzner, 2008; Beachboard and Horner, 2016; Chan and Gack, 2016; Lee et al., 2019). These strategies include sequestration or modification of viral nucleic acids, sequestration of PRRs, degradation or cleavage of host sensors or their adaptor proteins, interference with specific post-translational modifications of PRRs or their adaptor proteins, and inhibition of the enzymatic activity of PRRs. Evasion of the host innate immune response is crucial for herpesviruses to persist in their hosts. Therefore, understanding immune evasion strategies will advance our knowledge of viral/host interplay and viral pathogenesis in general, and will inform the development of preventive or therapeutic strategies against herpesviruses-associated diseases. In this review, we will discuss how human herpesviruses are sensed by the host innate immune system, with a focus on the DNA sensors, and elaborate the common evasion strategies that target different steps in this signaling pathway.

## Detection of Herpesviruses by PRRs

Herpesvirus virions contain a large, double-stranded DNA genome encased in a highly ordered icosahedral nucleocapsid. The nucleocapsid is coated with an amorphous layer known as tegument, consisting mostly viral proteins; which layer in turn is encased in a lipid bilayer envelope bearing distinct viral glycoproteins. Upon entry of the virus into host cells, some tegument proteins are released and the capsid is transported to the host nuclear membrane to deliver viral DNA into the nucleus. Transcription, replication of the viral genome, and assembly of the capsid take place in the nucleus (Boehmer and Nimonkar, 2003; Heming et al., 2017). The mature nucleocapsid egresses to the cytoplasm where it acquires tegument and envelope. The mature virions are transported through vesicles and finally released into the extracellular space. Alternatively, herpesviruses can maintain a latent state during which the viral genome is mostly silent and maintained as an episome in the nucleus with no progeny produced. The latent genome can be reactivated to initiate lytic replication upon certain cellular stress cues (Speck and Ganem, 2010; Cohen, 2020). The innate immune system is expected to recognize the components of the herpesvirus viral particles and replication intermediates produced during infection. Besides viral DNA, leaked mtDNA as a result of cellular stress from viral infection can also be sensed by DNA sensors such as cGAS (Paludan et al., 2011; Reinert et al., 2016; Sun et al., 2019).

### TLR9

**Figure 1:**
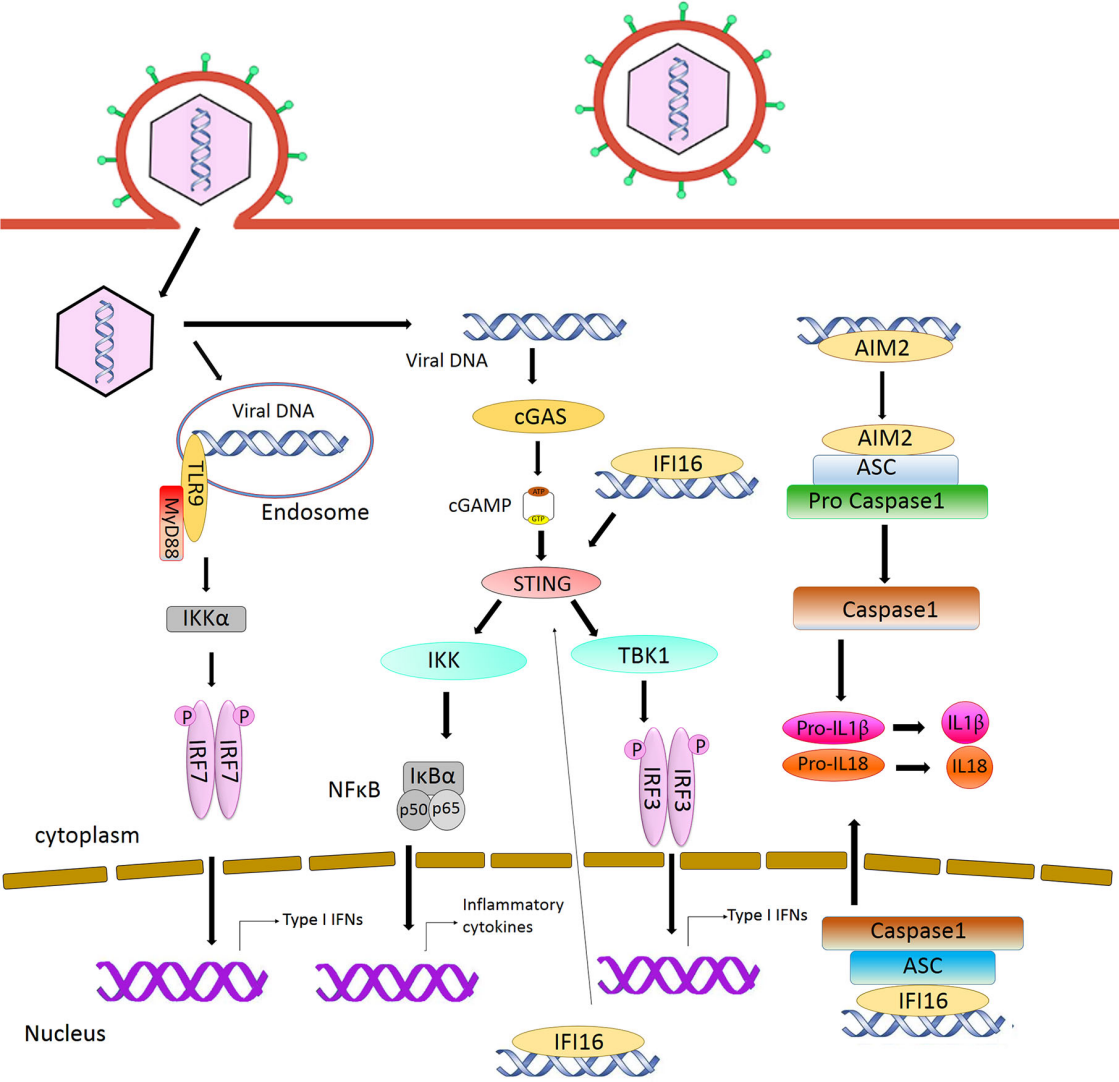
Intracellular sensing of herpesvirus DNA by DNA sensors and activation of signaling pathway. CpG DNA in the endosome is sensed by TLR9, leading to the production of type I interferon. AIM2 and IFI16 detect DNA in the cytoplasm and nucleus respectively and activate inflammasome pathway. Detection of DNA by cGAS activates STING signaling, resulting in the induction of type I interferon.

Toll-like receptors are the first characterized PRRs shown to recognize herpesviral PAMPs, including viral proteins, DNA and RNA. Among the TLRs, TLR2 detects virion components (Boehme et al., 2006; Leoni et al., 2012; Cai et al., 2013), while TLR3 and 9 recognize herpesvirus nucleic acid (Paludan et al., 2011; West et al., 2012). Specifically, TLR3 senses dsRNA (Iwakiri et al., 2009) and TLR9 detects endosomal dsDNA containing un-methylated CpG motifs that are commonly found in the herpesviral genome (Lund et al., 2003; Fiola et al., 2010). After DNA sensing, TLR9 recruits the adaptor protein MyD88 and induces the activation of Nuclear Factor κB (NF-κB) and Interferon Regulatory Factor (IRF)7, leading to the production of IFN-α in plasmacytoid Dendritic Cells (pDCs) (Kawai and Akira, 2011) (**Figure 1**). Infections from HSV-1, HSV-2, KSHV, and EBV have been shown to stimulate TLR9-mediated production of type I IFN in pDCs (Lund et al., 2003; Krug et al., 2004; Fiola et al., 2010; West et al., 2011; Ma and He, 2014). Infection from HSV-2 also induces a TLR9-mediated type III IFN response in DCs that relies more on NF-κB rather than IRFs (Iversen et al., 2010). HSV-induced activation of TLR9-mediated innate immune response is apparently cell-type-specific (Rasmussen et al., 2007). Krug et al. have shown that knockout of TLR9 causes no dramatic enhancement of HSV infection in mice (Krug et al., 2004), whereas another study reported that TLR9 expression in the trigeminal ganglia was required to prevent HSV encephalitis induced by intranasal HSV-1 infection, as TLR9 deficient mice were more susceptible to the virus, with 60% mortality (Lima et al., 2010).

### cGAS

The cyclic GMP–AMP synthase (cGAS) is the most recently discovered DNA sensor (Sun et al., 2013). It senses double-stranded DNA in a length-dependent but sequence-independent manner (Andreeva et al., 2017; Luecke et al., 2017). Binding to dsDNA leads to the formation of phase-separated liquid droplets in which cGAS is activated, and catalyzes the synthesis of cyclic GMP–AMP (cGAMP) from ATP and GTP (Du and Chen, 2018). As a second messenger, cGAMP activates ER-bound adaptor protein STING (STimulator of Interferon Genes) in the same cells or neighboring cells. Activated STING recruits kinase TBK1 and activates transcriptional factor IRF3 to initiate a downstream signaling cascade that culminates the expression of immune and inflammatory genes, such as type I IFNs (Tanaka and Chen, 2012) (**Figure 1**). Previously, it was thought that the cytoplasmic confinement of cGAS enabled it to specifically recognize pathogen- or damage-associated DNA. However, recent works revealed that cGAS is predominantly nuclear and its tight tethering to the chromatin is important in preventing its auto-reactivity (Volkman et al., 2019; Zierhut et al., 2019; Boyer et al., 2020; Kujirai et al., 2020; Michalski et al., 2020; Pathare et al., 2020; Zhao et al., 2020). The cGAS-cGAMP-STING pathway is crucial for host response to herpesviral infection. Sensing of HSV-1 DNA by microglia in the brain, and in PMA‐differentiated macrophage‐like THP1, induces type I IFN in a cGAS-STING dependent manner (Reinert et al., 2016), and cGAS deficient mice were found to be more susceptible to HSV-1 infection (Li X. D. et al., 2013; Christensen et al., 2016). Infection with HCMV was reported to activate the cGAS-STING pathway in primary Human Umbilical Vein Endothelial Cells (HUVEC) and in monocytic leukemia cell line THP-1 (Lio et al., 2016; Paijo et al., 2016). Although it has been reported that KSHV infection is also sensed by the cGAS-STING pathway and this pathway regulates the reactivation of KSHV from latency (Ma et al., 2015; Zhang et al., 2016), a recent report has shown that STING signaling is not critical in KSHV latent infection, replication, or in its spread after lytic reactivation in endothelial cells (Vogt et al., 2020).

### AIM2

Absent in melanoma 2 (AIM2) is a cytosolic DNA sensor that belongs to the PYHIN protein family. It contains an N-terminal Pyrin domain and one C-terminal HIN domain. The HIN domain binds to DNA through electrostatic interaction and the Pyrin domain associates with the adaptor protein ASC (apoptosis-associated speck-like protein containing a caspase activation and recruitment domain) through a homotypic PYRIN : PYRIN interaction. ASC further recruits pro caspase-1, leading to the production of active caspase-1. Caspase-1 in turn causes proteolytic maturation of the proinflammatory cytokines interleukin IL1β and IL18 (Hornung et al., 2009) (**Figure 1**). It has been shown that HCMV triggers the assembly of AIM2 inflammasome in THP-1-derived macrophages (Huang et al., 2017a; Botto et al., 2019). HSV-1 also induces AIM2-dependent inflammasome activation and IL-1β secretion in the absence of tegument protein VP22 in macrophages (Maruzuru et al., 2018).

### IFI16

Another member of the PYHIN family, IFI16, recognizes double-stranded DNA both in the cytosol and in the nucleus (Unterholzner et al., 2010). It contains an N-terminal Pyrin domain and two HIN domains in the C-terminus. The interaction with DNA takes place through the HIN domains. Similar to cGAS and AIM2, IFI16 also recognizes DNA in a length-dependent manner (Stratmann et al., 2015). IFI16 primarily localizes in the nucleus, where the DNA genome of large DNA virus resides and replicates. Kerur et al. showed for the first time that IFI16 acts as a nuclear DNA sensor of herpesvirus infection. Nuclear sensing of KSHV and EBV by IFI16 results in inflammasome responses through ASC and caspase-1, leading to the production of IL1β and IL18 (Kerur et al., 2011; Ansari et al., 2013; Johnson et al., 2013). IFI16 appears to interfere with HSV-1 replication. It has been shown that IFI16 binds to the HSV-1 genome at the transcription start sites of several viral genes, and blocks the recruitment of crucial cellular transcription factors to the promoters (Johnson et al., 2014). IFI16 was also found to be crucial for the maintenance of EBV latency (Pisano et al., 2017). It has been reported that IFI16 forms filamentous structure on DNA in the nucleus to promote the epigenetic silencing of viral DNA (Merkl and Knipe, 2019; Roy et al., 2019). Although IFI16 is predominantly nuclear, it can also detect DNA in the cytosol. During HSV-1 infection, IFI16 has been found to co-localize with viral genomic DNA in the cytoplasm. It is also probable that IFI16 binds to the viral genome in the nucleus and interacts with histone acetyltransferase p300. Acetylation of IFI16 results in its cytoplasmic redistribution, where it interacts with STING and subsequently induces IRF-3 phosphorylation as well as interferon-β production (Ansari et al., 2015). The stability of IFI16 is enhanced in the presence of the principal DNA sensor cGAS, and depletion of cGAS results in reduced expression of IL-6 transcript in a STING-independent manner (Orzalli et al., 2015). Recent studies have shown that IFI16 and cGAS both function cooperatively for the full activation of innate immune response to exogenous DNA (Orzalli et al., 2015; Almine et al., 2017; Jønsson et al., 2017).

### Other DNA Sensors

The first reported cytosolic DNA sensor was DNA-dependent activator of IRFs (DAI) (Takaoka et al., 2007). It detects DNA in the cytosol and activates type I IFNs through NF-κB and IRF3. Type I interferon expression in HCMV-infected fibroblast cells was reported to depend on DAI (Defilippis et al., 2010), which was shown to interact with receptor-interacting protein kinase (RIP)3 to arbitrate virus-induced necrosis during MCHV infection (Upton et al., 2012). DExD/H-box helicases DHX36 and DHX9 are the two sensors that detect CpG DNA and activate an MyD88-dependent pathway in pDCs (Kim et al., 2010). However, the interferon response and inflammatory response only partially depend on DHX36 and DHX9 respectively, presumably because TLR9 is the main sensor for CpG DNA. Several proteins involved in DNA damage repair can also serve as DNA sensors (Mboko et al., 2012). Meiotic recombination 11 homolog A (MRE11) and DNA-dependent protein kinase (DNA-PK) belong to that group. MRE11 also recognizes dsDNA and induces type I interferon (Kondo et al., 2013). DNA-PK is a heterotrimeric protein complex consisting of three subunits: Ku70, Ku80, and the catalytic subunit DNA-PKcs (Ferguson et al., 2012). It can exert antiviral responses in a STING-dependent and a STING-independent manner (Burleigh et al., 2020). The protein was shown to act as a DNA sensor and mediate the induction of type III interferon (Zhang et al., 2011). During HSV infection, cytokine response is impaired both in mice and in individual cells that are deficient in DNA-PK (Ferguson et al., 2012). Interestingly, DNA-PKcs activity has been previously reported to be degraded in an Infected Cell Protein 0 (ICP0) dependent manner in some cell types during HSV infection (Lees-Miller et al., 1996; Parkinson et al., 1999). The role of DNA-PK in sensing other herpesviruses is less clear, although EBV has been shown to trigger a DNA damage response (DDR) during both primary infection and lytic reactivation (Hafez and Luftig, 2017; Hau and Tsao, 2017). It has been recently shown that DNA damage results in the translocation of the principal DNA sensor cGAS to the nucleus, where it suppresses DNA repair and enhances cell proliferation. Thus cGAS appears to exhibit dual functions: as an innate immune sensor in the cytosol and as a negative regulator of DNA repair in the nucleus (Liu et al., 2018; Jiang et al., 2019).

## Evasion of DNA-Stimulated Immune Responses by Herpesviruses

The DNA genome of herpesvirus is shielded within the viral capsid until it reaches the nucleus. The nuclear DNA sensors IFI16 and hnRNP-A2B1 may recognize the viral DNA in the nucleus and translocate to the cytoplasm to elicit the immune response (Diner et al., 2015; Knipe, 2015; Wang et al., 2019). On the other hand, a defective virion could leak DNA into the cytoplasm that could be sensed by the cytosolic DNA sensors. This second notion is supported by observations that mutations altering capsid stability result in leakage of viral DNA in the cytoplasm and robust DNA sensing responses (Horan et al., 2013; Sun et al., 2019). Herpesvirus infection also induces mtDNA stress, which triggers antiviral responses (West et al., 2015). In order to persist in the hosts, viruses must overcome host immune defenses. Herpesviruses have evolved delicate strategies to avoid the host immune recognition. Here we will discuss evasion strategies (**Table 3**) that target either DNA sensors or their adaptor proteins to counteract the nuclear and cytoplasmic DNA sensing.

**Table 3:** Herpesvirus proteins that regulate DNA sensing and DNA activated signaling pathway.

	PRR	Virus	Viral protein	Experimental system	Proposed Mechanism	References
Inhibition of DNA sensing	IFI16	HSV-1	ICP0	human foreskin fibroblasts (HFF)	Promotes the 7 degradation of IFI16	Orzalli et al., 2012
			UL41	HFF, Hela	Degrades the *IFI16* mRNA	Orzalli et al., 2016
		HCMV	pUL83	HFF, human embryo kidney (HEK) 293T cell	Interacts with the IFI16 pyrin domain and blocks its oligomerization upon DNA sensing	Li T. et al., 2013
			pUL97	HELFs, HEK 293 cells	Binds to IFI16 and relocalizes it to the cytoplasm	Dell’oste et al., 2014
		KSHV	Lytic Proteins	BCBL-1 and BJAB cells	Promotes the ubiquitination and proteasomal degradation of IFI16	Roy et al., 2016
	cGAS	HSV-1	UL37	human monocyte THP-1 cells	Deamidates human and mouse cGAS	Zhang et al., 2018
			UL41	HEK293T	Selectively degrades cGAS mRNA	Su and Zheng, 2017
			VP22	HEK293T, Hela	Interacts with cGAS	Huang J. et al., 2018
		HCMV	UL31	HEK293T	Interacts with cGAS and disassociates DNA from cGAS	Huang Z. F. et al., 2018b
			pUL83	HFF, HEK 293	Binds to cGAS and inhibits its enzymatic activity	Biolatti et al., 2018
		KSHV	LANA	BCBL-1 PEL cell	Binds to cGAS	Zhang et al., 2016
			ORF52	*In Vitro* enzymatic assay, HEK293T–STING cells	Interacts with cGAS and DNA	Wu et al., 2015
		EBV	ORF52			
	AIM2	HSV-1	VP22	293FT cells, mouse macrophage cell line	Interacts with AIM2 and prevents its oligomerization	Maruzuru et al., 2018
			pUL83	HEK293T, macrophages	Interacts with AIM2 and inhibits activation of AIM2 inflammasome	Huang et al., 2017b
	DNA-pK	HSV-1	ICP0	HeLa S3 cells	Degrades the catalytic subunit of DNA-PK	Parkinson et al., 1999
		EBV	LMP1	nasopharyngeal carcinoma (NPC) cell line	Inhibits DNA-PK phosphorylation and activity	Lu et al., 2016
**Inhibition of DNA activated signaling pathway**	STING	HSV-1	ICP0	in HEp-2 cells	Stabilizes STING in HEp-2 cells, that is necessary for optimal HSV-1 replication	Kalamvoki and Roizman, 2014
			ICP27	THP 1 cells	Interacts with TBK1 and STING	Christensen et al., 2016
			UL46	HEK293T cells	Interacts with STING and TBK1	Deschamps and Kalamvoki, 2017; You et al., 2019
			γ_1_34.5	HFF	Binds to STING and perturbs its trafficking	Pan et al., 2018
		HCMV	UL42	HFF cell, HEK293T STING cell	Interacts with both cGAS and STING, inhibits cGAS-DNA interaction, oligomerization and enzymatic activity of cGAS. Impairs translocation of STING from the ER to perinuclear punctate structures.	Fu et al., 2019
			UL82	HFF	Interacts with and STING and inhibits its translocation from the ER to perinuclear microsomes	Fu et al., 2017
			pUL48	HFF	Deubiquitinase STING	Kumari et al., 2017
		KSHV	vIRF1	HEK293T STING cell	Prevents the phosphorylation and activation of STING by disrupting STING -TBK1 interaction	Ma et al., 2015

### Direct Inhibition of the Enzymatic Activity of the DNA Sensor

**Figure 2:**
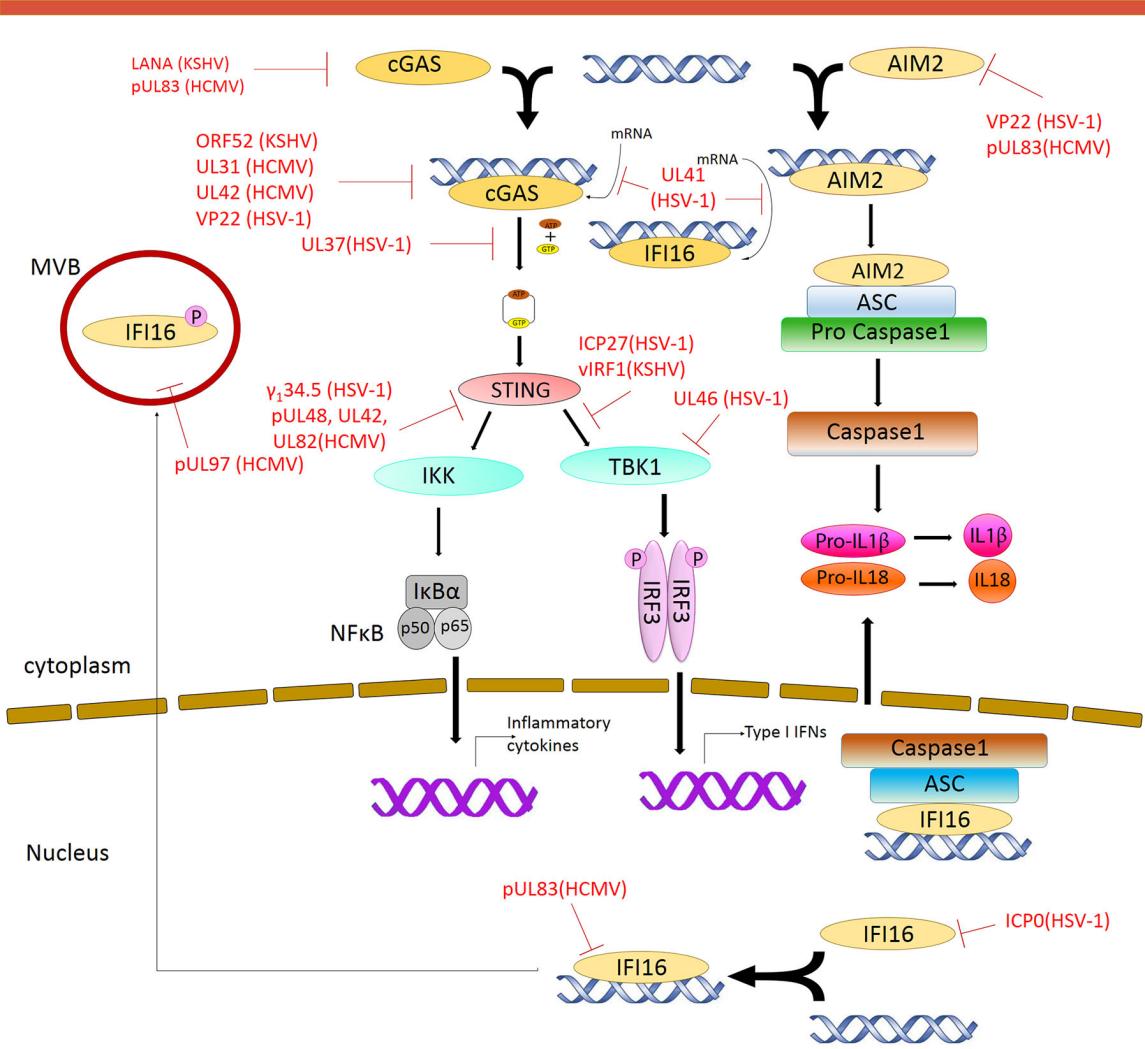
Modulation of DNA Sensing pathways by herpesvirus. Multiple steps in the signaling pathway are targeted by herpesvirus encoded proteins. Red solid line indicates inhibition of a particular pathway by the respective herpesvirus protein.

Viruses encode factors that can directly target the host DNA sensors to block their activation. Unique among the known DNA sensors, cGAS has catalytic activity and transmits signal through its enzymatic product cGAMP. Conceivably, interfering with the production of cGAMP or destroying cGAMP could be effective viral evasion strategies. While poxviruses and baculoviruses encode Poxin, a nuclease that degrades cGAMP, herpesviruses encode factors that directly inhibit cGAS activity. KSHV tegument protein ORF52 (KicGAS) was the first discovered inhibitor of cGAS enzymatic activity. KicGAS is a positively charged small and abundant tegument protein that binds to DNA efficiently. Purified KicGAS protein was shown to inhibit cGAS activity in a dose-dependent manner. This inhibition depends on its ability to bind DNA and cGAS. The inhibition of cGAS by KicGAS can be partially overcome by increasing the amount of DNA in the reaction, suggesting KicGAS uses a mechanism involving competition with DNA (Wu et al., 2015). In addition to KicGAS homologues in gamma herpesviruses, HSV-1 VP22, which exhibits limited structural homology to KicGAS, has also been reported to inhibit cGAS. Although no *in vitro* experimental data were shown, we suspect similar mechanisms were involved (Hew et al., 2015; Huang J. et al., 2018). Cytoplasmic isoforms of KSHV LANA (latency-associated nuclear antigen) interact with cGAS directly and block downstream signaling (Zhang et al., 2016). Two HCMV proteins, UL31 and UL42, interact with cGAS and interfere with cGAS-DNA binding (**Figure 2**) (Huang Z. F. et al., 2018; Fu et al., 2019). Another HCMV tegument protein, pUL83, selectively binds to cGAS and inhibits its enzymatic activity. The N-terminal domain of pUL83 appears to play an important role in binding, and the interaction is independent of viral DNA (Biolatti et al., 2018).

### Masking of the DNA Sensors

Another strategy used by the herpesvirus is to mask the DNA sensor to prevent downstream signaling. The DNA sensors that were shown to be sequestered by herpesviral proteins are IFI16 and AIM2. In addition to inhibit the enzymatic activity of cGAS, HSV-1 VP22 was also shown to interact with AIM2; the association is mediated by the HIN domain of AIM2. This interaction prevents the oligomerization of AIM2 (Maruzuru et al., 2018), which is the first step of AIM2-dependent inflammasome activation. The HCMV abundant virion protein pUL83 interacts with AIM2 and IFI16 in the cytoplasm and nucleus respectively (**Figure 2**) (Li T et al., 2013; Huang et al., 2017b). Interaction between pUL83 and AIM2 reduces the expression of inflammosome proteins and decreases the cleavage of caspase-1 and maturation of IL-1β. The protein pUL83 contains an N-terminal conserved pyrin *a*ssociation *d*omain (PAD) that interacts with the Pyrin domain of IFI16. It inhibits the nuclear oligomerization of IFI16 by sequestering the pyrin domain through the combined action of its conserved N- and C-terminals. Such oligomerization is essential for IFI16-mediated DNA-dependent immune signaling.

### Degradation of the DNA Sensors

In order to prevent activation of the DNA sensors, herpesviral proteins may target them for degradation. The DNA sensors IFI16, DNA-PK, RNF8, and RNF168 were shown to be degraded during herpesvirus infection. The ring finger domain of HSV-1 ICP0 exhibits E3 ubiquitin ligase activity. The nuclear form of the ICP0 protein was shown to interact with IFI16, relocalize IFI16 to ICP0-containing foci in the nucleus, and promote the proteasomal degradation of IFI16, resulting in impaired sensing of HSV-1 genome in HFFs (Orzalli et al., 2012; Johnson et al., 2013) (**Figure 2**). However, another study showed that ICP0 was neither required nor sufficient to promote the loss of IFI16 in U2OS and HepaRG cells (Cuchet-Lourenço et al., 2013), suggesting that the ICP0-mediated degradation of IFI16 could be cell-type specific. Studies also found IFI16 to be degraded during KSHV lytic replication, likely through the action of late protein(s) (Roy et al., 2016). ICP0 was reported to promote the proteasomal degradation of DNA-PK, RNF8, and RNF168, and modulate the DDR response (Parkinson et al., 1999; Lilley et al., 2011). The two ubiquitin ligases that anchor the repair factors at sites of damage (Parkinson et al., 1999; Chaurushiya et al., 2012).

In addition, viral proteins can also promote the selective degradation of host mRNA and reduce the level of the DNA sensors cGAS and IFI16. The HSV-1 tegument protein UL41 was shown to selectively degrade cGAS mRNA *via* its mRNA-specific RNase activity, reduce the level of cGAS, and downregulate cGAS STING-mediated signaling (Su and Zheng, 2017). UL41 was also reported to contribute to the reduction of IFI16 levels (Orzalli et al., 2016).

### Post-Translational Modification of the DNA Sensors

DNA sensing is also regulated by post-translational modifications including phosphorylation, ubiquitination, acetylation, sumoylation, and glutamylation of the DNA sensors (Wu and Li, 2020). Acetylation and phosphorylation of IFI16 at different sites have been shown to regulate its subcellular localization (Li et al., 2012). HCMV viral protein kinase pUL97 phosphorylates IFI16 and triggers its relocalization from nucleus to cytoplasm, away from the HCMV genome in the nucleus, thus inhibiting nuclear sensing of viral DNA (Dell’oste et al., 2014). However, ectopic expression of pUL97 alone was insufficient to relocate IFI16, suggesting additional HCMV components may be involved. HSV-1 tegument protein UL37 deamidates a critical Asp in the activation loop of human and mouse cGAS, resulting in impaired cGAMP synthesis (Zhang et al., 2018) (**Figure 2**). It was previously demonstrated that DNA binding leads to conformation change in the activation loop of cGAS that is required for its catalytic activity (Zhang et al., 2014). The deamination of Asp in the activation loop presumably blocks the conformational change. It is to be noted that this Asp is not conserved in non-human primates, thus HSV-1 mediates species specific inactivation of cGAS.

### Interfering With the Downstream Signaling Pathway

Most DNA sensors transmit signals through the adaptor protein STING, which possesses both interferon-dependent and interferon-independent immune responses (Wu et al., 2020; Yamashiro et al., 2020). After activation, STING translocates from the ER to endosomal/lysosomal perinuclear regions where it associates with the kinase TBK1 (Ishikawa and Barber, 2008). This interaction mediates the activation of transcription factors IRF3 and (NF-κB), and promotes the expression of immune and inflammatory genes, such as type I IFNs (Tanaka and Chen, 2012). In order to antagonize the STING-mediated immune response, herpesvirus encodes a number of proteins that directly target STING for deubiquitination, perturb its intracellular trafficking, or block its interaction with other signaling partners (Ahn and Barber, 2019; Liu et al., 2019; Yang et al., 2019). HSV-1 protein pUL36 directly associates with and deubiquitinate STING and perturbs its downstream signaling (Bodda et al., 2020). HCMV protein pUL48 was also reported to deubiquitinate STING (Kumari et al., 2017). The HSV-1 protein γ_1_34.5, and HCMV tegument proteins UL42 and UL82, were shown to impair STING trafficking from the ER to the Golgi apparatus. Protein UL82 interacts with STING and ER-associated protein iRhom2, and disrupts the iRhom2-mediated formation of the STING-TRAPβ translocon complex (Fu et al., 2017). On the other hand, UL42 does not interact with iRhom2. It promotes the degradation of TRAPβ and inhibit STING trafficking (Fu et al., 2019). But how HSV-1 γ_1_34.5 blocks STING signaling is not clearly understood (Pan et al., 2018). The proteins vIRF1 of KSHV and ICP27 and UL46 of HSV-1, were shown to disrupt STING-TBK1 interaction. Interaction between vIRF1 and STING inhibits the STING-mediated phosphorylation of TBK1 (Ma et al., 2015). But ICP27 interacts with the STING-TBK1 signalosome and inhibits the phosphorylation of IRF3 by TBK1 (Christensen et al., 2016). In addition, UL46 interacts with STING and TBK1 *via* its N- and C-termini respectively and impairs the activation of IRF3 by inhibiting the dimerization of TBK1 (Deschamps and Kalamvoki, 2017; You et al., 2019). Interestingly, ICP0 of HSV-1 was found to stabilize STING in certain cell types such as HEp-2 cells, as it is needed for optimal HSV-1 infection in those cells (Kalamvoki and Roizman, 2014). In addition, herpesviral proteins also target STING interaction partners to attenuate innate immune response downstream of STING (Christensen and Paludan, 2017; Liu et al., 2019). Thus, herpesvirus evolved mechanisms targeting various steps of DNA sensing to block immune response.

## Conclusion and Perspectives

With the discovery of a number of DNA sensors over the past decade, significant progress has been made on understanding the host innate immune response to herpesviruses. Along with this, our understanding on how herpesviruses target different steps of this signaling pathway to establish persistent infection has greatly expanded. However, there are still several key questions that need to be answered. First, if most of the DNA sensors recognize DNA in sequence independent manner, then what is the need of so many DNA sensing pathways, and what are their relative contributions to *in vivo* DNA sensing? One possibility is that the presence of multiple DNA sensing pathways in different cellular compartments may provide multiple opportunities for the innate immune recognition of aberrant DNA (Emming and Schroder, 2019). Different DNA sensors may require different ligand (DNA concentration) thresholds for their activation. So, it may be helpful for the cell to fine-tune the immune response based on the escalating level of danger imposed by viral infection. It remains a major challenge to understand how these pathways act in concert to detect and elicit cell-type–specific or species-specific responses to DNA. Second, nuclear sensors are hypothesized to bind to unchromatinized viral DNA to initiate innate signaling, but viral DNA is chromatinized upon entry into the nucleus. Therefore, the process of nuclear DNA sensing and the time frame between entry and the chromatinizaton of viral DNA is crucial and needs further investigation. Future efforts are necessary to better understand how nuclear-originating immune signaling is transmitted to the cytosol and back to the nucleus. Recently it has been shown that the dsDNA sensor heterogeneous nuclear ribonucleoprotein A2B1 (hnRNP-A2B1) recognizes viral dsDNA in the nucleus, then dimerizes and translocates to the cytosol to initiate type 1 interferon response *via* TBK1-IRF3 pathway (Wang et al., 2019). Further studies on the molecular mechanism by which the host nuclear DNA sensors such as hnRNP-A2B1 discriminate viral DNA from self genomic DNA will be critical for understanding nuclear DNA sensing. Third, a number of immune evasion strategies have been discovered recently, but the detailed mechanisms remain to be elucidated in most cases. Better understanding of the viral immune evasion mechanisms should aid in the development of vaccines and antivirals against herpesviruses.

In addition, herpesviruses can cause long-lasting infection as a result of the mutualistic equilibrium between the ability of the virus to survive under host antiviral immunity and the ability of the host to tolerate the presence of the virus continuously (Cruz-Muñoz and Fuentes-Pananá, 2018). The mutualistic equilibrium is not always beneficial to the host. It may lead to disease characterized by the abnormality in the immune system. Moreover, there is a significant overlap between the PRRs that sense herpesvirus and the PRRs involved in autoimmune disease. Further investigation in this area is necessary to identify the missing link between herpesvirus and autoimmune disease that could be useful to reduce unwanted inflammation.

## Author Contributions

DB and FZ wrote the manuscript and designed the figures. All authors contributed to the article and approved the submitted version.

## Funding

This work is funded by NIH grants R01 DE026101 to FZ.

## Conflict of Interest

The authors declare that the research was conducted in the absence of any commercial or financial relationships that could be construed as a potential conflict of interest.
